# Supplementary material for: Extracellular Calcium-Induced Calcium Transient Regulating the Proliferation of Osteoblasts through Glycolysis Metabolism Pathways
Source: Int J Mol Sci. 2023 Mar 5;24(5):4991. doi: 10.3390/ijms24054991 (PMC10003245; doi:10.3390/ijms24054991)
Supplement: Supplementary file 1 [file ijms-24-04991-s001.zip › ijms-2190879-supplementary.pdf]

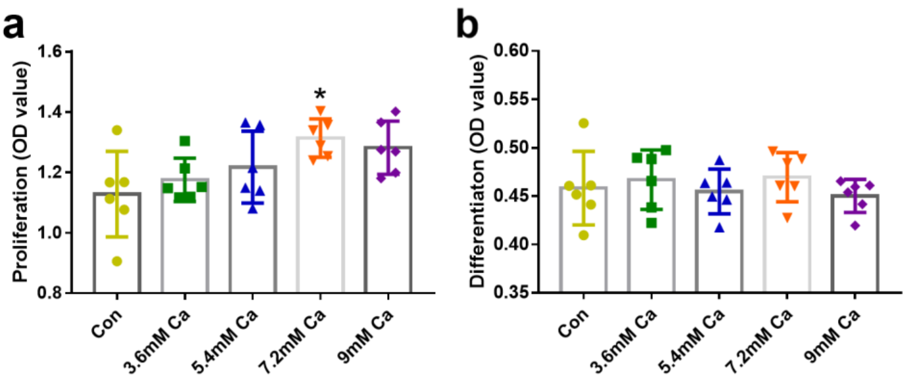

Figure S1. (a) Proliferation of osteoblast cells induced by different concentration-response of the extracellular Ca^{2+} P. *$P < 0.05$, *vs*. Con group. (b) Differentiation of osteoblast cells induced by different concentration-response of the extracellular Ca^{2+}.
